# Analysis of the Aging-Related AP2/ERF Transcription Factor Gene Family in *Osmanthus fragrans*

**DOI:** 10.3390/ijms25158025

**Published:** 2024-07-23

**Authors:** Gongwei Chen, Tianqi Shao, Yixiao Zhou, Fengyuan Chen, Dandan Zhang, Heng Gu, Yuanzheng Yue, Lianggui Wang, Xiulian Yang

**Affiliations:** Key Laboratory of Landscape Architecture, College of Landscape Architecture, Nanjing Forestry University, No. 159 Longpan Road, Nanjing 210037, China; chengongwei0118@163.com (G.C.); 838631072@njfu.edu.cn (T.S.); 13767185707@163.com (Y.Z.); chenfengyuan@njfu.edu.cn (F.C.); d2273429918@126.com (D.Z.); guheng@njfu.edu.cn (H.G.); yueyuanzheng@njfu.edu.cn (Y.Y.); wlg@njfu.com.cn (L.W.)

**Keywords:** transcriptome, *Osmanthus fragrans*, flower senescence

## Abstract

Ethylene-Responsive Factor (ERF) is a key element found in the middle and lower reaches of the ethylene signal transduction pathway. It is widely distributed in plants and plays important roles in plant growth and development, hormone signal transduction, and various stress processes. Although there is research on AP/ERF family members, research on AP2/ERF in *Osmanthus fragrans* is lacking. Thus, in this work, AP2/ERF in *O. fragrans* was extensively and comprehensively analyzed. A total of 298 genes encoding *OfAP2/ERF* proteins with complete AP2/ERF domains were identified. Based on the number of AP2/ERF domains and the similarity among amino acid sequences between AP2/ERF proteins from *A. thaliana* and *O. fragrans*, the 298 putative *OfAP2/ERF* proteins were divided into four different families, including AP2 (45), ERF (247), RAV (5), and SOLOIST (1). In addition, the exon–intron structure characteristics of these putative *OfAP2/ERF* genes and the conserved protein motifs of their encoded *OfAP2/ERF* proteins were analyzed, and the results were found to be consistent with those of the population classification. A tissue-specific analysis showed the spatiotemporal expression of *OfAP2/ERF* in the stems and leaves of *O. fragrans* at different developmental stages. Specifically, 21 genes were not expressed in any tissue, while high levels of expression were found for 25 *OfAP2/ERF* genes in several tissues, 60 genes in the roots, 34 genes in the stems, 37 genes in young leaves, 34 genes in old leaves, 32 genes in the early flowering stage, 18 genes in the full flowering stage, and 37 genes in the late flowering stage. Quantitative RT-PCR experiments showed that *OfERF110a* and *OfERF*110b had the highest expression levels at the full-bloom stage (S4), and this gradually decreased with the senescence of petals. The expression of *OfERF119c* decreased first and then increased, while the expression levels of *OfERF4c* and *OfERF5a* increased constantly. This indicated that these genes may play roles in flower senescence and the ethylene response. In the subsequent subcellular localization experiments, we found that ERF1-4 was localized in the nucleus, indicating that it was expressed in the nucleus. In yeast self-activation experiments, we found that *OfERF112*, *OfERF228*, and *OfERF23* had self-activation activity. Overall, these results suggest that *OfERFs* may have the function of regulating petal senescence in *O. fragrans.*

## 1. Introduction

The gaseous plant hormone ethylene is involved in many aspects of plant growth and development [1]. The aging of flowers is related to an increase in ethylene production in flowers, and endogenous ethylene has been shown to play a regulatory role in events leading to the death of certain flower organs [2]. According to the DNA-binding domain protein sequence, the Ethylene-Responsive Factor (ERF) family is mainly divided into four subfamilies: DREB (dehydration response element binding), ERF (ethylene response element binding protein), AP2 (APETALA2), RAV (associated with ABI3/VP), and Soloists (a few unclassified factors) [3,4]. In addition, a variety of AP2/ERF transcription factors have been successfully identified and studied in some plants, including rice (*Oryza sativa*) [5], grape (*Vitis vinifera*) [6], poplar (*Populus tricocarpa*) [7], wheat (*Triticum aestivum*) [8], cucumber (*Cucumis sativus*) [9], barley (*Hordeum vulgare*) [10], and soybean (*Glycine max*) [11].

In plants, the APETALA2/ERF (AP2/ERF) superfamily is one of the largest plant transcription factor families, and 174 AP2/ERF family members have been found in *Arabidopsis thaliana* under cold stress conditions [12]. A total of 209 ERF transcription factor genes have been found in poplar [13]. AP2/ERF superfamily proteins contained at least one highly conserved AP2/ERF DNA binding domain, including 50–60 DNA binding domains and amino acid residues. According to the number and similarity of AP2/ERF DNA binding domains, they can be divided into four different families; namely, the AP2 family, the ERF family (including two subfamilies, ERF and DREB), the RAV family, and the Soloists family [14] (Table S4). AP2 family members have been shown to play important roles in regulating the development of nutrition and reproductive organs [15]. In *A. thaliana*, studies have shown that the overexpression of *AtERF019* may delay the senescence of flowers [16]. In petunia, ERF transcription factors have been associated with flower senescence [17]. Similarly, the ERF transcription factor is known to be an important factor in petunia petal senescence [18].

*O. fragrans* is an important ornamental plant and an evergreen woody flower of the Oleaceae family and the Osmanthus genus. It is also a traditional Chinese plant which is widely used in classical Chinese gardens. Due to its rich aroma and color, which represent wealth, it is still loved by people today. However, *O. fragrans* has a short flowering period that is concentrated in September and October. In adverse weather conditions, the flowering period would be further shortened. Therefore, studying the aging of *O. fragrans* flowers and prolonging its flowering period is of great significance.

Therefore, in this study, a comprehensive analysis of the AP2/ERF family was conducted based on previously publicly available whole-genome data for *O. fragrans* to provide important information for understanding the function and evolution of the AP2/ERF gene family in *O. fragrans*.

## 2. Results

### 2.1. Identification Results for AP2/ERF Transcription Factor Family Members in O. fragrans

A total of 298 AP2/ERF transcription factors were predicted in the genome of *O. fragrans*. According to the sequence number in the genome and the naming rules for *A. thaliana* AP2/ERF family members, the screened genes were named *OfERF*1-*OfERF*247, *OfAP2-1-OfAP2-45*, *OfRAV1-5*, and *OfSOLO1*. The online analysis results showed that the length of the AP2/ERF transcription factor protein was 59–684 aa, the molecular weight was 11.86–75.49 kDa, the theoretical isoelectric point prediction range was 4.27–11.33, and the instability index was 22.23–81.67. The hydrophilicity index was negative, indicating that these proteins were hydrophilic proteins (Table S1).

### 2.2. Phylogenetic Analysis of AP2/ERF Transcription Factor Family Members in O. fragrans

**Figure 1:**
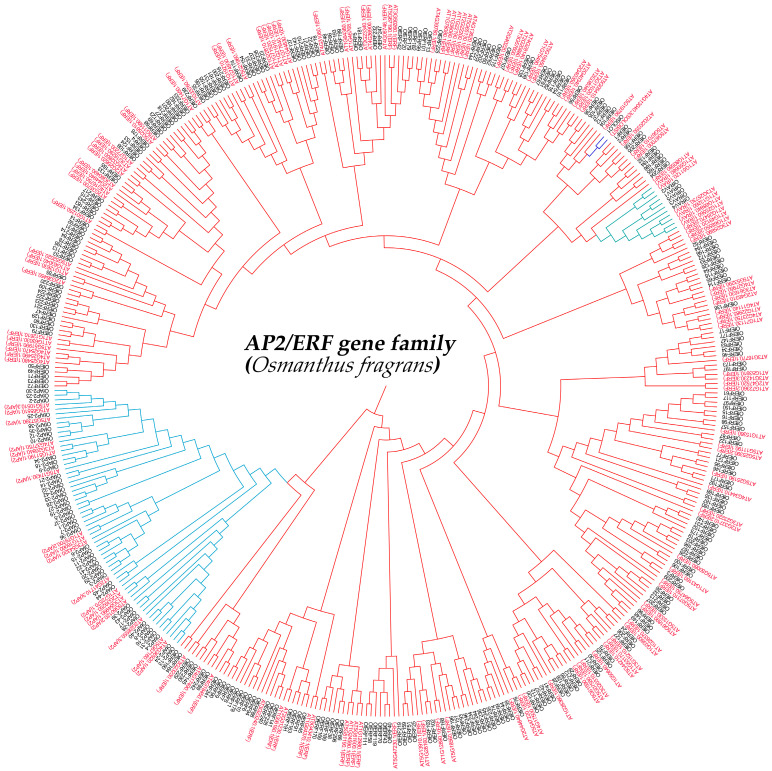
Phylogenetic analysis of AP2/ERF transcription factors of *A. thaliana* and *Osmanthus fragrans*. Red represents AP2/ERF factors in *A. thaliana*; black represents AP2/ERF factors in *O. fragrans*.

The phylogenetic tree results showed that the subclasses of *A. thaliana* AP2/ERF members basically clustered together, with *O. fragrans* and *A. thaliana* AP2/ERF members of the same types being very close. The analysis of the number of AP2/ERF family members in *O. fragrans* showed that the AP2/ERF transcription factor family members can be divided into AP2 (45), ERF (247), RAV (5), and SOLOIST (1) (Figure 1).

### 2.3. Analysis of the Gene Structure, Conserved Domain, and Subcellular Localization of AP2/ERF Transcription Factors in O. fragrans

By comparing the CDS sequence and genomic DNA sequence, it was found that the number of exons in the 298 genes ranged from 1 to 9. All members of the OfAP2/ERF family were found to have a conserved AP2 domain, of which 247 *OfERF* members contained a typical AP2 domain. Of the 45 OfAP2 members, 42 contained two AP2 domains, and 3 contained only one. Five OfRAV members contained one AP2 and one B3 domain. Among the transcription factors, 273 were distributed in the nucleus, 16 in the chloroplasts, and 9 in the mitochondria (Table S1).

**Figure 2:**
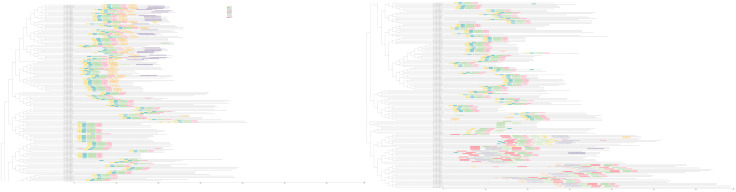
Conserved motifs in the protein sequences.

**Figure 3:**
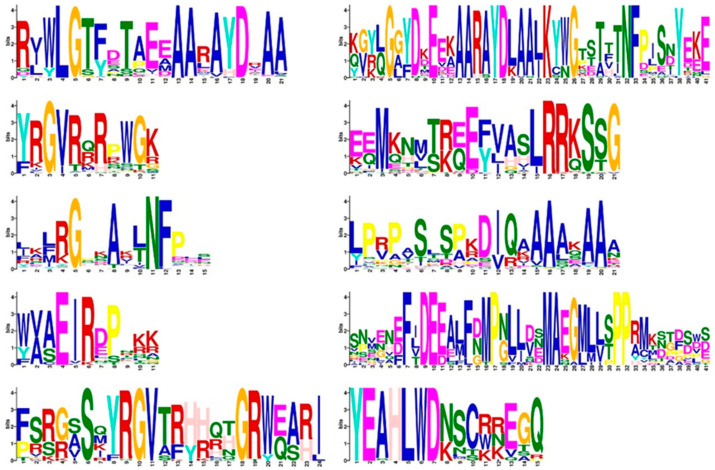
Conserved motif analysis for the protein family members.

In addition, 10 different motifs were identified, named motifs 1–10, of which motif 1 appeared in all family members (Figure 2). In the OfERF subfamily, most members contained motif 2–3–1–8 in order, indicating that this domain was more conserved in this subfamily. In the OfAP2 subfamily, most members contained sequential motifs 2–9–5–4, indicating that this domain was more conserved in this subfamily. In the OfRAV subfamily, each member contained the ordered motif 2–1, indicating that this domain was more conserved in this subfamily (Figure 3).

### 2.4. Analysis of Chromosomal Localization and Duplication Events within AP2/ERF Transcription Factors in O. fragrans

**Figure 4:**
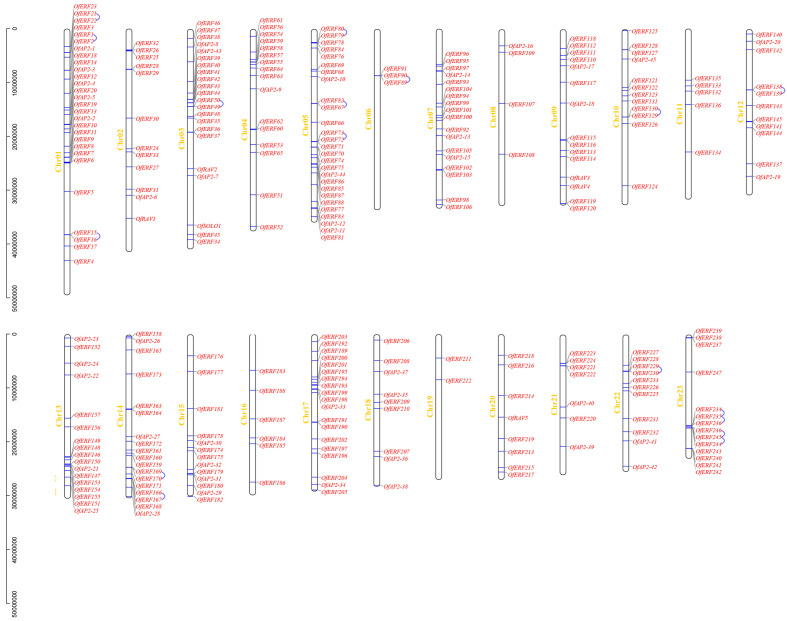
Chromosomal distribution of AP2/ERF genes in *O. fragrans*. Blue lines represent tandem repeats.

The 298 genes were found to be distributed on 23 chromosomes of *O. fragrans*, of which Chr19 had the lowest distribution, with a total of 2. Chr01 had the highest distribution, with a total of 28. OfAP2/ERF was more distributed at the upper ends of Chr01, 03, 04, and 17. It was more distributed at the lower ends of Chr13, 14, and 15. There was a high distribution density at both ends of Chr05 and 23 (Figure 4).

**Figure 5:**
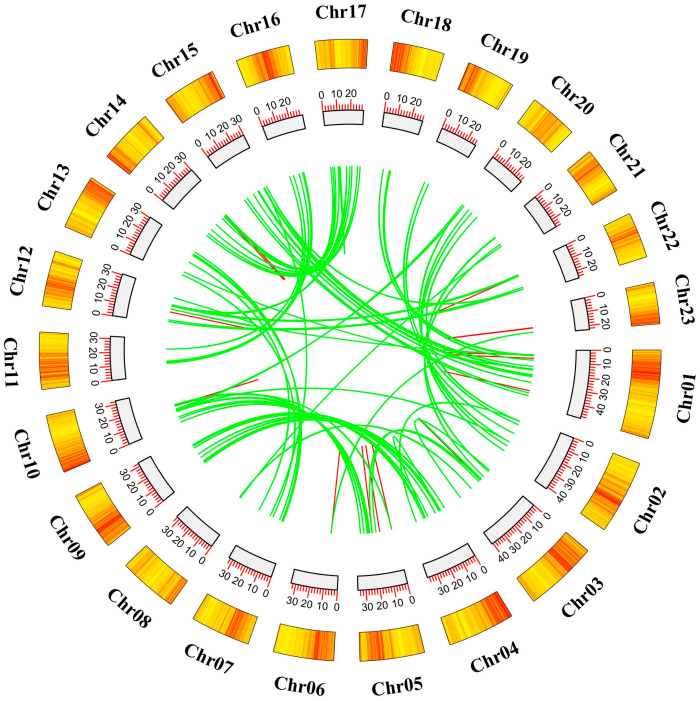
Duplicated AP2/ERF gene pairs in the *O. fragrans* genome. Green lines represent duplicated genes, blue lines represent tandem repeats.

Tandem gene duplication is a gene duplication event that occurs in two or more homologous genes on the same chromosome. Fragment duplication is a homologous gene duplication event between different chromosomes. From Figure 4, it can be seen that there are 17 pairs of tandem repeat genes in the OfAP2/ERF gene family, involving 32 genes, which are distributed on chromosomes Chr01, 03, 05, 06, 10, 12, 14, 22, and 23. Among them, Chr23 has the highest distribution, with four pairs, involving six genes. The genes with tandem repeats belong to the same branch on the phylogenetic tree, and the sequence similarity is high, which further proves the reliability of the gene distribution on the phylogenetic tree. Through the analysis and drawing of Circos in Tbtools, 91 pairs of fragment replication events were found in OfAP2/ERF. The highest fragment replication frequency occurred between Chr04 and Chr09, including 12 fragment replication pairs; the second highest was between Chr01 and Chr15, with a total of 10 fragment repeat pairs (Figure 5).

### 2.5. Expression Analysis Results for the AP2/ERF Gene in O. fragrans

**Figure 6:**
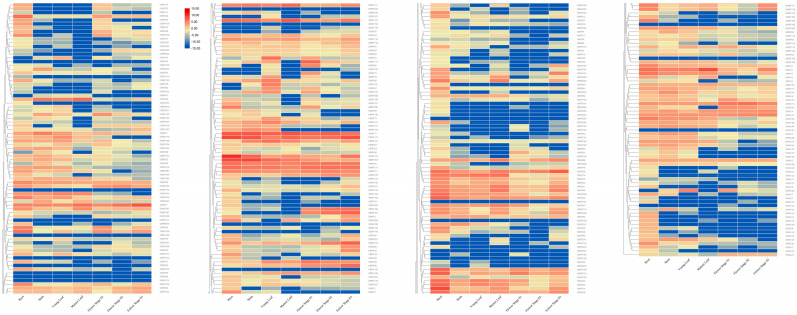
Expression patterns of AP2/ERF genes in different tissues of *O. fragrans*.

The visual expression profile (Figure 6) showed that 21 genes were not expressed in any tissues, indicating that they were pseudogenes or expressed under certain conditions. The 25 *OfAP2/ERF* genes were highly expressed in several tissues, indicating that these genes may play important regulatory roles in different developmental stages of *O. fragrans*. There were 60 genes with higher expression in roots, 34 genes with higher expression in stems, 37 genes with higher expression in young leaves, 34 genes with higher expression in old leaves, 32 genes with higher expression in the early flowering stage, 18 genes with higher expression in the full flowering stage, and 37 genes with higher expression in the late flowering stage.

**Figure 7:**
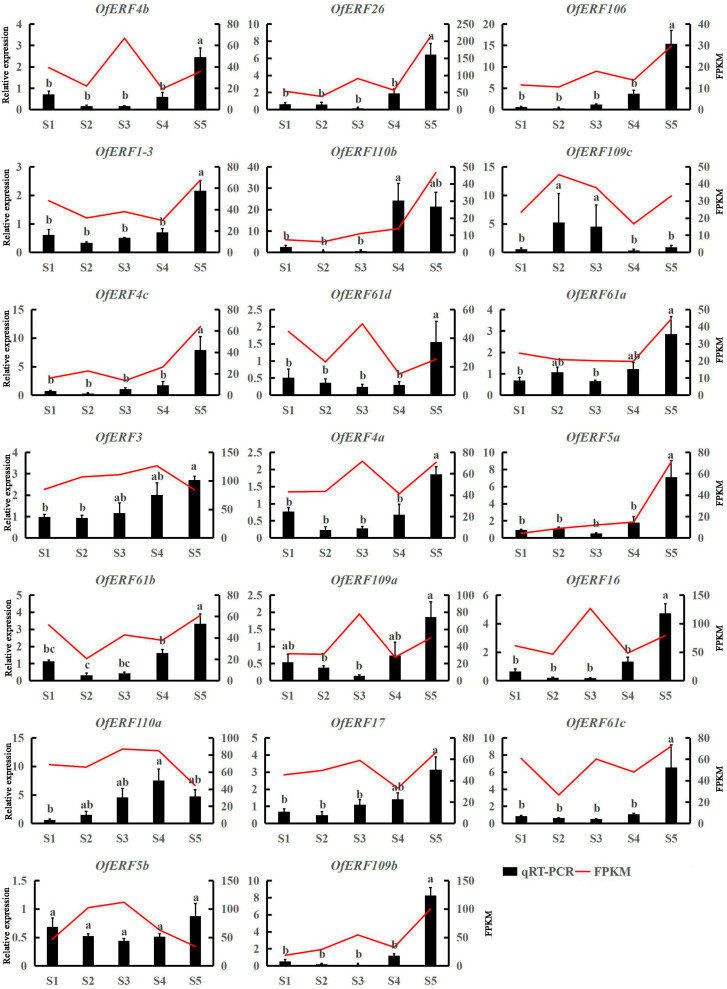
Expression patterns of AP2/ERF genes at different flowering stages of *O. fragrans*. S1: Linggeng stage, S2: Xiangyan stage, S3: initial flowering stage, S4: full flowering stage, S5: late flowering stage. ‘a, b’ are part of the gene name.

According to the expression profile analysis and transcriptome data, 20 genes with high expression and differential expression in the five flowering stages were selected for qRT-PCR verification (Figure 7). The results show that *OfERF1-3*, *3*, *4a*, *4b*, *4c*, *5a*, *5b*, *16*, *17*, *26*, *61a*, *61b*, *61c*, *61d*, *106*, *109a*, and *109b* had the highest expression levels in the late flowering stage (S5). The expression levels of *OfERF110a* and *110b* were the highest at the full-bloom stage (S4) and gradually decreased during petal senescence. During the gradual senescence of *O. fragrans* petals (S3–S4–S5), the expression trends of *OfERF119c*, *OfERF4c*, and *OfERF5a* were consistent with the expression trends of transcriptome data. Among them, *OfERF119c* showed a trend of decreasing first and then increasing, while the expression levels of *OfERF4c* and *OfERF5a* were always increasing.

### 2.6. The Result of Yeast Self-Activation Verification and Subcellular Localization

**Figure 8:**
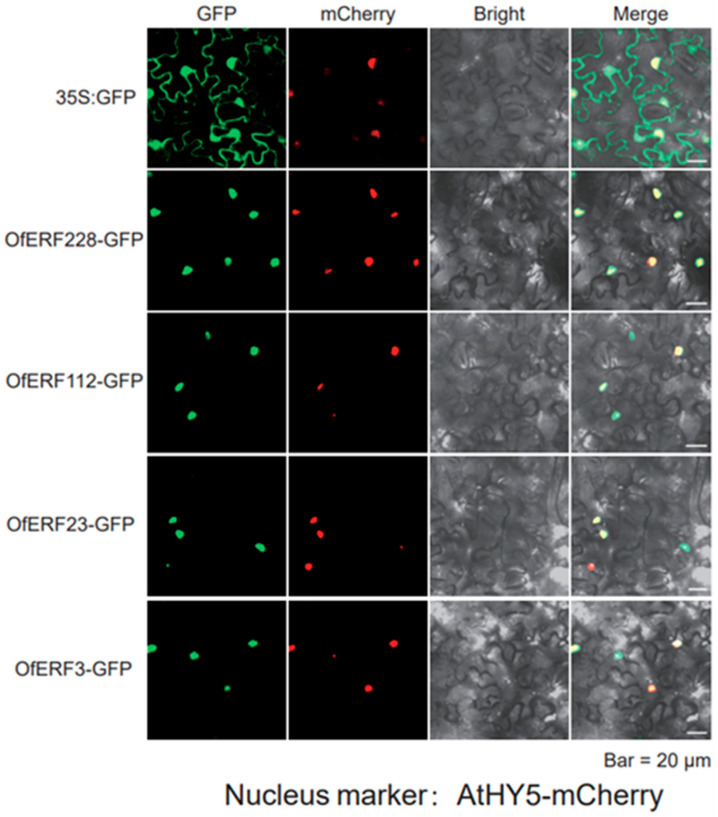
Subcellular localization of the OfERFs-GFP gene.

The OfERFs-GFP members were found to be co-localized with nuclear localization markers, indicating that these OfERFs proteins were localized in the nucleus (Figure 8).

**Figure 9:**
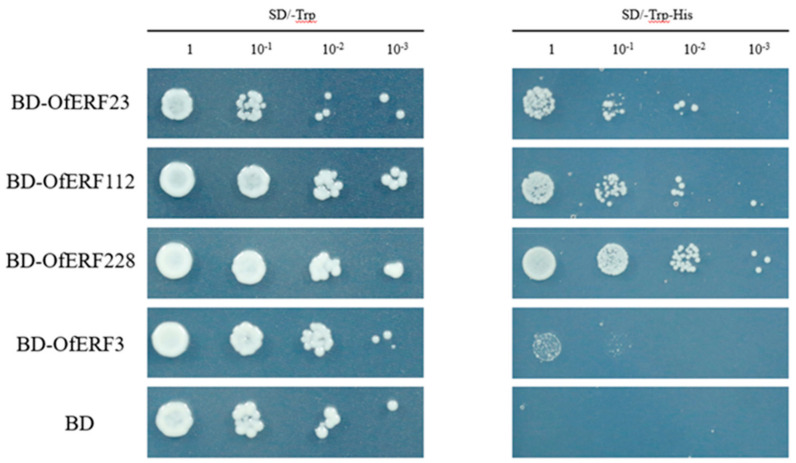
Yeast self−activation verification.

The yeast-based transcriptional activity assays showed that three out of the five OfAP2/ERF proteins grew well on SD Trp and SD Trp his media, indicating a protein transactivation activity. The growth of OfERF3 was similar to that in the negative control, indicating that these proteins may not function as transcriptional activators in the yeast heterologous system (Figure 9).

## 3. Discussion

The AP2/ERF gene family is one of the largest gene families in plants [19]. The first AP2/ERF gene was discovered in *A. thaliana*. The family plays important roles in plant growth and development, stress resistance, and flower senescence. Although AP/ERF family members have been extensively studied, research on these genes in *O. fragrans* is lacking. Therefore, in this work, the characteristics of the AP2/ERF family in *O. fragrans* were extensively and comprehensively analyzed. 

Transcription factors control the expression of downstream genes in signal transduction pathways through post-stress activation or inhibition [1]. Studies have shown that AP2/ERF transcription factors have multiple functions in plant growth and development, and a large number of genes are involved in plant hormone response pathways and flower development processes [20]. So far, there have been few reports on members of the AP2/ERF gene family in *O. fragrans*. Therefore, the identification of *O. fragrans* AP2/ERF gene family members can help us understand the potential functions of *O. fragrans* genes.

### 3.1. Expression Analysis Results for the AP2/ERF Gene in O. fragrans

Comparison of species homologs, including sequenced genomes and expression profiles, may help us understand the roles of these transcription factors in *O. fragrans*. We assume that transcriptional regulators within the same taxon exhibit recent co-evolutionary origins and specific conserved motifs associated with molecular function. This hypothesis can be used as an effective and practical way to predict the functions of unknown proteins, which are derived from the structural relationships of *A. thaliana*. In this study, the AP2/ERF amino acid sequence of *A. thaliana* was used as a probe. By comparing the genomic protein sequence of *O. fragrans*, 298 AP2/ERF gene family members were identified in *O. fragrans*, with the number of genes being higher than those of *A. thaliana* (147) [4], rice (*Oryza sativa*) (163) [5], maize (*Zea mays*) (167) [21], grape (*Vitis vinifera*) (145) [6], and poplar (*Populus alba*) (142) [7]. Gene duplication events continue to occur with the evolution of plants, resulting in an increase in gene family members and the derivation of new gene functions. The number of genes in the AP2/ERF family of many species expands due to gene replication events [22]. In *O. fragrans*, we identified 17 tandemly repeated AP2/ERF genes and 91 fragmentally repeated AP2/ERF genes, suggesting that the evolution of the AP2/ERF gene family in *O. fragrans* may also be driven by gene replication events. According to the results of the phylogenetic analysis and conserved domain analysis, *OfAP2/ERF* genes were divided into four subfamilies, *OfAP2*, *OfRAV*, *OfERF*, and *OfSOLO*, which was consistent with previous studies.

Transcription factor domains and motifs are usually associated with protein–protein interactions, transcriptional activity, and DNA binding [23]. In addition, AP2/ERF superfamily genes have similar characteristics in both dicots and monocots [24]. *O. fragrans* genes mainly contained AP2-domain-related genes named motifs 1–10, among which motif 1 appeared in all family members. In the OfERF subfamily, most members contained motifs 2-3-1-8. In the OfAP2 subfamily, most members contained the sequential motifs 2-9-5-4. In the OfRAV subfamily, each member contained the ordered motif 2-1. The conserved motif analysis showed that these conserved motifs may be closely related to their functions, such as their transcriptional functions [25]. Further, in soybean and Arabidopsis, these conserved domains have been shown to be associated with ethylene-related transcription factors. Among them, the conserved motif ‘RVWLGTFDTAEEAARAYDEAA’ was considered to be a conserved domain related to the ethylene transcription factor, the conserved motif of ‘SNMENEFLDEEAMFNMPGLLDSMAEGMLLTPPAMKNGFSWS’ was considered to be a dehydration response element in soybean [26], and the conserved motif in ‘WAAEIRDPTRK’ was confirmed to be related to stress resistance in soybean. These conserved motifs may have the same functions in *O. fragrans* [11]. However, the functions of these motifs remained to be further elucidated, and more work was needed to be conducted to explore their regulatory functions. Taken together, these results suggested that the emergence of these conserved motifs may play a crucial functional role in the evolution of diverse organisms. 

Further, most members of the AP2/ERF family have no introns, and *O. fragrans* is no exception with most of the 303 genes identified lacking introns. This typical gene structure pattern was consistent with previous studies on *A. thaliana* [25], jujube (*Ziziphus jujuba)* [27], and grape (*Vitis vinifera*) [28]. For example, in *A. thaliana*, 90 genes have no introns. Studies have shown that the number and distribution of introns are related to the evolution of plants [29]. In addition, studies have shown that the loss of introns in genes after fragment replication occurs faster than the acquisition of introns [30]. The evolutionary rate of species may be related to the loss of introns in genes, so we speculate that the loss of introns in *O. fragrans* may also be related to the evolution of *O. fragrans*.

### 3.2. OfERF qRT-PCR and Tissue Specificity

Duplication of a single gene, chromosome segment, or entire genome has long been considered a major source of evolution, including new gene functions and expression patterns. Our study showed that most of the duplicated *OfERF* genes were expressed in different tissues/organs, and 25 *OfAP2/ERF* genes were highly expressed in several tissues, indicating that these genes may play important regulatory roles in different developmental stages of *O. fragrans*. ERF family genes showed higher expression levels in these tissues, which may be due to the low content of ERF family introns. Due to the small number of introns, ERF family genes can respond faster and exhibit higher expression levels during development [24]. In plant organs, the most genes were expressed in roots (60) and showed the lowest expression in the full-bloom stage (18). About 35 genes were expressed in other organs. This may indicate that OfAP2/ERF transcription factors are involved in plant metabolite biosynthesis and trait development [31]. We also found that 21 genes were not detected; these may be pseudogenes, or some of these genes may be involved in biotic and abiotic stress processes and may not be detected [32]. In vegetative organs, the transcripts of these genes were most abundant in roots, followed by leaves and stems; in reproductive tissues, high expression of the family genes was observed in embryos and lemmas.

The ERF transcription factors identified in this study were unevenly distributed on 23 chromosomes of *O. fragrans*. Subcellular localization prediction showed that the ERF gene was mainly located in the nucleus. In addition, the comparative analysis of expression patterns in different tissues showed that the expression of AP2/ERF family members differed among different tissues and organs such as roots, stems, leaves, and flowers. These results will contribute to the functional analysis of AP2/ERF family genes in *O. fragrans* in the future.

### 3.3. Gene Function of OfERFs

ERF is a key element in the middle and lower reaches of the ethylene signal transduction pathway. It is widely distributed in plants and plays an important role in plant growth and development, hormone signal transduction, and various stress processes [14]. The *A. thaliana* AP2 homologous gene had a certain expression intensity in all four-wheeled flower tissues during flower development and was involved in the regulation of floral organ formation [33]. In this study, 20 ERF transcription factors were analyzed using qRT-PCR. Most of the genes (17) had the highest expression at the late flowering stage (S5), indicating that they may be related to flower senescence. Among them, two genes had the highest expression level at the full-bloom stage (S4), gradually decreasing during petal senescence. Similarly, in petunia, *ERF1* was shown to be associated with flower senescence [17], and was found to activate senescence-related genes and promote flower senescence in *A. thaliana* [34]. *ERF1-3* was also found to have the function of silencing genes in *A. thaliana* [35]. Further, *ERF1* was found to regulate the aging process in waterlily [36]. This is consistent with the increase in the expression of most *ERF1* genes at the late flowering stage (S) shown in this study, which may indicate that *ERFs* in *O. fragrans* were also involved in flowering senescence regulation. Further, some studies have shown that ERF may be related to stress resistance because ERF is also a key regulator of various stress responses, and a regulatory network related to ERF and hormones were found in *A. thaliana* [37]. For example, *ERF5a* may be an antioxidant-enzyme-coding gene and a stress response gene [38]. *ERF16* may be a transcriptional activator of LIPOXYGENASE D, a key gene in jasmonic acid biosynthesis, allene oxide cyclase, and 12-oxo phytodienoate reductase 3 [39]. *ERF17* may be involved in the regulation of CML5 and H2S [40]. ERF26 may be related to fruit preservation [41]. *ERF109a* may be related to salt stress [42]. However, further experiments and research are needed to verify these hypotheses.

We selected five transcription factors for further analysis, of which *OfERF23*, *112*, and *228* had obvious self-activation activity, while *OfERF3* had weak self-activation activity. *LrAP2/ERF23* may be a gene related to anthocyanin synthesis in *Lycoris radiata* [43]. *OsERF112* is a key regulator of bacterial leaf blight resistance in rice [44]. It was also found that the interaction between rice *ERF3* and *WOX11* can promote growth of the crown and root, suggesting that they may be involved in the regulation of cytokinin signal transduction [45]. In *O. fragrans*, these genes may have the same function, but this hypothesis needs further experimental verification.

## 4. Materials and Methods

### 4.1. Experimental Materials

The test material used was *O. fragrans* ‘Rixianggui’, sampled from a mature *O. fragrans* tree on the campus of Nanjing Forestry University. The height and age of the sampling tree were 3–4 m and about 8–10 a, respectively. In this experiment, samples of roots, stems, leaves, and flowers were collected. All samples were immediately frozen in liquid nitrogen for 10 min after collection and then stored in an ultra-low-temperature refrigerator for later use. The development of *O. fragrans* ‘Rixiang’ roughly involves the linggen stage (S1), budeye stage (S2), early flowering stage (S3), full flowering stage (S4), and late flowering stage (S5).

Transcriptome sequencing samples were derived from the roots, stems, and leaves (young and mature leaves) from the same tree and flowers at five different developmental stages. For each sample, three biological replicates were used. Total RNA was extracted using the TRNzol universal kit (Tiangen, Beijing, China). The quantity and quality of RNA were evaluated using an Agilent Bioanalyzer 2100 (Agilent Technologies, Santa Clara, CA, USA), a NanoDrop™ One UV-Vis spectrophotometer (Thermo Fisher Scientific, Waltham, MA, USA), and a Qubit^®^ 3.0 fluorometer (Thermo Fisher Scientific, Waltham, MA, USA). The transcriptome sequencing results of roots, stems, leaves, and flowers (S1–S5) of *O. fragrans* were published at NCBI (PRJNA932144).

### 4.2. Identification of AP2/ERF Transcription Factor Family Members in the O. fragrans Genome

The AP2/ERF transcription factor of *O. fragrans* was identified from the previously published whole-genome database of *O. fragrans* [46]. According to the hidden Markov model PF00847 from the PFAM database, the HMMER search was performed for the *O. fragrans* genome database, and the Evalue was set to 1 × 10^−5^ [47,48]. In addition, the protein sequences of the AP2, ERF, RAV, and SOLOIST members of the AP2/ERF family in *A. thaliana* were used for a BLASTP comparison with the genomic protein sequence of *O. fragrans*, and the Evalue was set to 1 × 10^−5^. After the search was completed, all *O. fragrans* AP2/ERF sequences were extracted from the genome database gff file via Tbtools according to the searched sequence number [49]. After extraction, the CDS, DNA, and protein sequence information for each AP2/ERF member were saved. HMMSCAN www.ebi.ac.uk/Tools/hmmer/search/hmmscan (accessed on 20 October 2020) and NCBI Conserved Domain Search Service (CDSearch) www.ncbi.nlm.nih.gov/Structure/bwrpsb/bwrpsb.cgi (accessed on 22 October 2020) were used to confirm the integrity of the conserved domain sequence of each member. The number of amino acids, molecular weight (MW), and theoretical isoelectric point (pI) of each AP2/ERF member were obtained using the online ProtParam tool https://web.expasy.org/protparam/ (accessed on 2 December 2020).

### 4.3. Phylogenetic Analysis of AP2/ERF Family Members in O. fragrans

In order to study the evolutionary relationship of the AP2/ERF transcription factor family, the full-length protein sequences of 147 *A. thaliana* and 298 *O. fragrans* AP2/ERF members were used to construct a phylogenetic tree using MEGAX 5.2 software. The protein sequences of *A. thaliana* AP2/ERF (AtAP2/ERF) family members were derived from the PlnTFDB database. The MUSCLE program in MEGAX 5.2 software was used to perform multiple sequence alignments on the protein sequences of the AP2/ERF members of *O. fragrans* and *A. thaliana*, and then the neighbor joining (NJ) method was used to construct the phylogenetic tree. The number of repetitions was set to 1000. Finally, FigTree v1.4.2 http://tree.bio.e.ac.uk/ (accessed on 20 October 2020) software was used to draw the phylogenetic tree image.

### 4.4. Gene Structure, Conserved Domain, Conserved Motif, and Subcellular Localization Analysis

The gene structure details of the AP2/ERF transcription factor were obtained from the *O. fragrans* genome database GFF3 file. The gene structure of *OfAP2/ERF* was predicted using TBtools V1.045 software combined with GFF3 file information and the ID number of *OfAP2/ERF*. The Pfam tool (http//pfam.sanger.ac.uk/) was used to analyze and confirm all conserved domains identified. Finally, TBtools software was used to draw the gene structure information and the conserved domain distribution map [49]. A subcellular localization analysis of AP2/ERF transcription factors was performed using the subcellular localization prediction software CELLO v.2.5 http//cello.life.nctu.edu.tw (accessed on 20 November 2020). The MEME program http://meme-suite.org/tools/meme (accessed on 8 November 2020) was used to obtain 10 conserved protein motifs, and the optimal motif width was set to 6–200.

### 4.5. Chromosomal Localization, Gene Replication, and Evolutionary Analysis

MapInspect 1.0 software was used to extract the locations of AP2/ERF transcription factor chromosomes from the *O. fragrans* genome database, and graphics were drawn. Synteny analysis of AP2/ERF transcription factors was performed using the Quick MCScanX program in TBtools, and the collinearity analysis map was drawn with the Amazing Super Circos in TBtools V1.045 software [50]. Finally, the AP2/ERF transcription factors were mapped using the Circos tool, and their replication relationships on 23 chromosomes were plotted.

### 4.6. De Novo Assembly of RNA-Seq Reads and Quantification of Gene Expression

In order to ensure the quality of data, it is necessary to filter the original data before information analysis so as to reduce the analysis interference caused by invalid data. First of all, we used fastp [51] https://github.com/OpenGene/fastp (accessed on 20 October 2020) for quality control, filtering low-quality data (remove reads with adapter, remove reads with N ratio greater than 10%, remove all A-base reads, remove the quality value Q ≤ 20 base number accounts for more than 50% of the whole read) to obtain clean reads. Then, the spliced reads in RNA Seq sequencing data were compared with HISAT2 [52] using global and local search methods. For each transcription region, an FPKM (fragment per kilobase of transcript per million mapped reads) value was calculated to quantify its expression abundance and variations using StringTie v1.3.6 software.

### 4.7. AP2/ERF Gene Expression Analysis

Based on publicly available whole genome information of *O. fragrans* and transcriptome data of *O. fragrans* ‘Rixianggui’ [46], we evaluated the expression levels of the *OfAP2/ERF* genes in roots (young and mature), stems, leaves, and flowers at three developmental stages (early flowering, full flowering, and late flowering) and generated heat maps using TBtools V1.045 software based on RPKM value levels [50]. Twenty key genes were selected for qRT-PCR verification. The PCR primers of the gene were designed using Primer5 software (Table S3). *OfRAN* was selected as the internal reference gene [53]. An RNA extraction kit (Aidlab Biotechnologies Co., Ltd., Beijing, China) was used to extract RNA from five flowering stages of *O. fragrans*, DNA was removed by DNA removal column, and the liquid was lysed by RLT. RNA concentration and quality were further determined using an accounting detector, where A260/A280 for each sample ranged from 1.8 to 2.2. RNA extracted from *O. fragrans* leaves was reverse transcribed into cDNA using SuperMix (Transgen, Beijing, China) and diluted 20 times for gene expression test. The cDNA of each qRT-PCR sample was 1 μL. The qRT-PCR reaction kit used was the SYBR^®^ Premix Ex Taq™ (Takara) kit (Wuhan, China). The reaction procedure is as follows: reaction at 95 °C for 30 s; stored at 95 °C for 5 s; 60 °C, 30 s, 40 cycles. The dissolution curve program is as follows: 95 °C, 15 s, 60 °C, 1 min, 95 °C, 15 s [53]. Each sample was subjected to three biological replicates and three technical replicates. SPSS v.24 (IBM, Armgonk, NY, USA) analysis of variance (ANOVA) was used to test whether there was a significant difference in the experiment, and Duncan’s multiple range test (*p* < 0.05) was used to test whether there was a significant difference between the samples.

### 4.8. Subcellular Localization and Yeast Self-Activation

For the subcellular localization experiment, *Agrobacterium tumefaciens* solution was added to YEP culture medium containing kanamycin and rifampicin. This was followed by shaking at 28 °C and 200 rpm to achieve an OD600 of 0.6–1.0, and bacterial cells were collected via centrifugation. The buffer was resuspended with *A. tumefaciens* (10 mM MES (pH 5.7)), 10 mM MgCl_2_, and 200 μL MAS of resuspended bacterial cells, and the OD600 was adjusted to 0.1–0.2. Then, samples were left at room temperature for 1–4 h. Next, different infection liquids were mixed in equal volumes and injected into the leaves of Honji tobacco. After 2–3 days of cultivation in the incubator, the fluorescence signal was observed using a laser confocal microscope and photos were taken.

For the yeast self-activation experiment, yeast receptive cells (Y2H Gold) (oebiotech, Shanghai, China) were removed from the −80 °C refrigerator and thawed on ice. The PEG/LiOAc master mix was prepared by adding 1.2 mL of 50% PEG, 180 μL of 1 M LiOAc, and 125 μL of fish sperm DNA while mixing. For every 100 μL, 0.5 μg of plasmid was added to the receptive cells and 300 μL PEG/LiAc to the master mix. This was followed by vortex mixing, placement in a 42 °C water bath for 45 min, and then vortexing and mixing every 15 min at 700× *g* 5 min. Then, 150 μL of the supernatant was removed. After resuspending with 0.9% NaCl solution, the plate was applied, and cultivation occurred at 30 °C. Following the above steps, the BD plasmid containing the target gene was transferred into the Y2Hgold yeast receptive state and applied to SD Trp screening solid culture medium, and the 30 °C incubator was inverted for 2 days. Then, three monoclonal clones were selected and placed in SD Trp liquid on a 30 °C shaker overnight. Next, 2.5 μL points were taken at four gradient concentrations of 1, 10-1, 10-2, and 10-3 and inverted in SD Trp and SD Trp/His solid culture medium for 2–3 days. The reactions were observed and photos were taken.

The vectors for subcellular localization and yeast self-activation were Super 1300 and pGBKT7, respectively. The restriction sites of the gene were selected by BioXM 2.6 software and the amplification primers of the overexpression vector were designed. The subcellular localization and yeast self-activation control genes were named as 35S: GFP and pGBKT7-EV, respectively.

## 5. Conclusions

In summary, we found that *OfEREs* have similarities in gene structure and function with ERFs found in other species. We conducted qRT-PCR analysis and other experiments on *OfERFs*, and found that the expression level of *OfERFs* in each period of *O. fragrans* flowers had a significant trend. According to the research in other species, it was speculated that *OfERFs* should have the function of regulating the senescence of *O. fragrans*.

## Data Availability

No new data were created or analyzed in this study. Data sharing is not applicable to this article.

## Supplementary Materials

The following supporting information can be downloaded at: https://www.mdpi.com/article/10.3390/ijms25158025/s1.
