# Supplementary material for: Analysis of the Aging-Related AP2/ERF Transcription Factor Gene Family in Osmanthus fragrans
Source: Int J Mol Sci. 2024 Jul 23;25(15):8025. doi: 10.3390/ijms25158025 (PMC11312093; doi:10.3390/ijms25158025)
Supplement: Supplementary file 1 [file ijms-25-08025-s001.zip › Table S1.pdf]

Table S1. Gene name of each OfERF family

Name	Gene ID	Number of amino acids / per	Molecular weight / kDa	IEP	Instability inde	Hydrophilicity index	Subcellular localization
OfERF1	evm.model.Contig106.32	264	29.35	5.98	39.58	-0.573	Nucleus
OfERF2	evm.model.Contig106.33	315	35.21	6.84	52.13	-0.649	Nucleus
OfERF3	evm.model.Contig109.138	338	37.09	5.84	64.43	-0.854	Nucleus
OfERF4	evm.model.Contig11.363	205	22.09	5.16	58.34	-0.409	Nucleus
OfERF5	evm.model.Contig163.2	139	15.63	9.33	60.63	-0.874	Nucleus
OfERF6	evm.model.Contig166.31	177	19.90	5.02	59.54	-0.851	Nucleus
OfERF7	evm.model.Contig166.36	179	20.20	4.86	66.34	-0.730	Nucleus
OfERF8	evm.model.Contig166.39	174	19.32	5.05	57.29	-0.684	Nucleus
OfERF9	evm.model.Contig166.44	174	19.34	4.91	61.87	-0.730	Nucleus
OfERF10	evm.model.Contig215.79	177	19.91	5.02	60.63	-0.850	Nucleus
OfERF11	evm.model.Contig215.82	179	19.99	4.84	60.04	-0.684	Nucleus
OfERF12	evm.model.Contig223.146	384	43.35	4.60	51.38	-0.807	Nucleus
OfERF13	evm.model.Contig231.60	333	37.11	5.47	58.08	-0.555	Nucleus
OfERF14	evm.model.Contig247.44	161	18.29	5.67	39.76	-0.511	Chloroplast
OfERF15	evm.model.Contig6.128	177	19.97	7.69	59.18	-0.729	Nucleus
OfERF16	evm.model.Contig6.129	176	19.98	8.93	65.50	-0.803	Nucleus
OfERF17	evm.model.Contig6.233	391	42.98	4.80	38.59	-0.716	Nucleus
OfERF18	evm.model.Contig60.156	362	41.43	5.78	49.61	-0.869	Nucleus
OfERF19	evm.model.Contig66.2	377	41.44	8.81	66.27	-0.628	Nucleus
OfERF20	evm.model.Contig71.167	164	18.03	9.63	34.52	-0.671	Nucleus
OfERF21	evm.model.Contig84.166	231	25.04	7.59	53.03	-0.149	Chloroplast
OfERF22	evm.model.Contig84.167	234	25.30	5.86	54.97	-0.254	Nucleus
OfERF23	evm.model.Contig84.64	141	15.70	5.44	51.79	-0.507	Nucleus
OfERF24	evm.model.Contig135.89	212	23.03	9.22	53.91	-0.669	Nucleus
OfERF25	evm.model.Contig34.13	240	26.30	5.67	48.32	-0.418	Nucleus
OfERF26	evm.model.Contig34.6	228	25.56	5.56	52.18	-0.928	Nucleus
OfERF27	evm.model.Contig346.84	247	26.43	5.32	52.47	-0.347	Nucleus
OfERF28	evm.model.Contig366.2	159	17.40	9.51	44.45	-0.586	Mitochondria

OfERF29	evm.model.Contig40.1	157	17.24	9.51	44.76	-0.568	Mitochondria
OfERF30	evm.model.Contig42.42	227	24.69	6.74	63.05	-0.644	Nucleus
OfERF31	evm.model.Contig45.65	218	23.50	6.30	54.10	-0.445	Nucleus
OfERF32	evm.model.Contig466.1	228	25.56	5.56	52.18	-0.928	Nucleus
OfERF33	evm.model.Contig554.11	147	16.21	5.84	52.05	-0.388	Nucleus
OfERF34	evm.model.Contig281.4	287	32.10	7.07	29.38	-0.789	Nucleus
OfERF35	evm.model.Contig312.6	206	22.98	4.99	54.93	-0.564	Nucleus
OfERF36	evm.model.Contig319.18	322	36.92	4.85	68.22	-0.901	Nucleus
OfERF37	evm.model.Contig319.20	378	42.77	4.74	59.96	-0.855	Nucleus
OfERF38	evm.model.Contig342.1	326	36.23	8.97	49.11	-0.651	Nucleus
OfERF39	evm.model.Contig35.190	172	19.17	9.35	45.99	-0.563	Chloroplast
OfERF40	evm.model.Contig35.191	290	32.81	6.94	44.93	-0.834	Nucleus
OfERF41	evm.model.Contig35.193	214	24.13	8.23	63.39	-0.721	Nucleus
OfERF42	evm.model.Contig35.282	214	24.13	8.23	63.39	-0.721	Nucleus
OfERF43	evm.model.Contig35.283	290	32.83	6.99	44.93	-0.833	Nucleus
OfERF44	evm.model.Contig35.284	172	19.17	9.35	45.99	-0.563	Chloroplast
OfERF45	evm.model.Contig352.1	321	36.53	8.41	50.21	-0.582	Nucleus
OfERF46	evm.model.Contig501.13	287	32.06	6.34	28.14	-0.793	Nucleus
OfERF47	evm.model.Contig629.11	206	23.11	6.52	53.32	-0.540	Nucleus
OfERF48	evm.model.Contig64.26	273	30.91	8.83	43.65	-0.620	Nucleus
OfERF49	evm.model.Contig64.273	224	24.62	5.34	48.42	-0.440	Chloroplast
OfERF50	evm.model.Contig64.274	198	21.97	5.45	58.53	-0.469	Chloroplast
OfERF51	evm.model.Contig152.73	252	28.44	5.06	45.25	-0.950	Nucleus
OfERF52	evm.model.Contig246.25	352	39.05	4.66	43.76	-0.661	Nucleus
OfERF53	evm.model.Contig274.14	204	22.30	4.61	69.10	-0.679	Nucleus
OfERF54	evm.model.Contig33.126	368	41.27	5.67	36.49	-0.943	Nucleus
OfERF55	evm.model.Contig33.24	188	19.94	7.84	59.43	-0.605	Nucleus
OfERF56	evm.model.Contig33.293	208	23.37	5.71	33.37	-0.824	Nucleus
OfERF57	evm.model.Contig33.76	225	25.38	5.52	65.65	-0.627	Nucleus
OfERF58	evm.model.Contig33.77	228	25.80	6.46	45.23	-0.674	Nucleus
OfERF59	evm.model.Contig33.78	244	27.05	8.72	65.83	-0.453	Chloroplast
OfERF60	evm.model.Contig370.6	320	33.93	9.30	56.57	-0.626	Nucleus

OfERF61	evm.model.Contig53.182	186	20.88	5.72	68.75	-0.678	Nucleus
OfERF62	evm.model.Contig619.14	318	33.72	9.30	54.65	-0.618	Nucleus
OfERF63	evm.model.Contig92.244	289	32.07	5.54	44.59	-0.758	Nucleus
OfERF64	evm.model.Contig92.68	342	38.66	4.95	42.65	-0.821	Nucleus
OfERF65	evm.model.Contig95.88	361	40.73	5.18	49.23	-0.867	Nucleus
OfERF66	evm.model.Contig105.116	274	31.07	8.44	49.22	-0.510	Nucleus
OfERF67	evm.model.Contig118.82	335	37.00	5.21	44.82	-0.789	Nucleus
OfERF68	evm.model.Contig122.95	223	24.09	5.29	61.34	-0.485	Nucleus
OfERF69	evm.model.Contig149.87	223	24.10	5.29	60.48	-0.461	Nucleus
OfERF70	evm.model.Contig170.10	274	31.12	7.76	50.52	-0.641	Nucleus
OfERF71	evm.model.Contig170.100	174	18.99	8.93	46.75	-0.509	Nucleus
OfERF72	evm.model.Contig170.102	227	24.84	5.11	49.56	-0.461	Nucleus
OfERF73	evm.model.Contig170.103	227	25.12	4.96	51.73	-0.468	Chloroplast
OfERF74	evm.model.Contig170.8	234	25.87	6.77	57.25	-0.552	Nucleus
OfERF75	evm.model.Contig207.97	234	25.69	8.56	63.24	-0.765	Nucleus
OfERF76	evm.model.Contig221.32	164	19.00	11.33	55.56	-0.708	Mitochondria
OfERF77	evm.model.Contig235.45	59	6.91	10.24	32.85	-0.797	Nucleus
OfERF78	evm.model.Contig336.3	180	19.45	4.81	48.41	-0.301	Nucleus
OfERF79	evm.model.Contig336.4	208	23.00	5.08	56.13	-0.543	Nucleus
OfERF80	evm.model.Contig336.5	239	26.59	5.72	47.34	-0.501	Nucleus
OfERF81	evm.model.Contig410.30	340	37.51	5.19	45.64	-0.758	Nucleus
OfERF82	evm.model.Contig434.27	284	31.54	7.56	51.21	-0.785	Nucleus
OfERF83	evm.model.Contig54.43	363	40.68	5.83	51.21	-0.764	Nucleus
OfERF84	evm.model.Contig583.20	232	25.13	5.60	55.47	-0.509	Nucleus
OfERF85	evm.model.Contig78.177	159	17.31	9.74	50.06	-0.440	Mitochondria
OfERF86	evm.model.Contig78.192	131	14.47	9.72	55.29	-0.520	Nucleus
OfERF87	evm.model.Contig78.96	215	23.84	9.23	52.02	-0.749	Nucleus
OfERF88	evm.model.Contig86.32	203	23.10	6.13	53.50	-0.572	Nucleus
OfERF89	evm.model.Contig114.146	125	14.24	5.46	22.23	-0.861	细胞质
OfERF90	evm.model.Contig114.148	138	15.71	5.83	73.41	-1.013	Nucleus
OfERF91	evm.model.Contig114.149	179	19.79	9.02	47.23	-0.420	Chloroplast
OfERF92	evm.model.Contig148.88	222	24.13	9.12	52.23	-0.320	Chloroplast

OfERF93	evm.model.Contig183.4	329	36.45	4.79	53.80	-0.732	Nucleus
OfERF94	evm.model.Contig189.56	139	15.84	8.97	44.59	-0.681	Nucleus
OfERF95	evm.model.Contig191.100	162	18.53	7.66	64.00	-0.765	Nucleus
OfERF96	evm.model.Contig191.133	299	33.98	9.68	67.21	-0.708	Nucleus
OfERF97	evm.model.Contig191.7	187	20.68	9.33	68.60	-0.628	Nucleus
OfERF98	evm.model.Contig224.22	198	22.55	6.92	75.94	-0.856	Nucleus
OfERF99	evm.model.Contig257.54	332	36.98	8.46	49.05	-0.384	Nucleus
OfERF100	evm.model.Contig262.40	294	31.29	5.66	52.47	-0.471	Nucleus
OfERF101	evm.model.Contig262.8	332	37.08	8.66	49.67	-0.396	Nucleus
OfERF102	evm.model.Contig263.3	220	24.20	5.35	60.48	-0.612	Nucleus
OfERF103	evm.model.Contig307.25	220	24.20	5.49	61.16	-0.625	Nucleus
OfERF104	evm.model.Contig507.37	271	29.99	7.02	48.31	-0.577	Nucleus
OfERF105	evm.model.Contig571.12	172	19.37	9.30	57.27	-0.673	Nucleus
OfERF106	evm.model.Contig587.4	294	31.32	5.66	51.68	-0.478	Nucleus
OfERF107	evm.model.Contig133.58	284	32.36	6.55	57.06	-0.901	Nucleus
OfERF108	evm.model.Contig140.76	232	25.61	5.16	52.57	-0.518	Nucleus
OfERF109	evm.model.Contig204.80	216	22.72	4.27	59.59	-0.312	Nucleus
OfERF110	evm.model.Contig190.42	372	41.48	5.24	48.31	-0.835	Nucleus
OfERF111	evm.model.Contig313.3	239	27.39	7.73	61.00	-0.740	Nucleus
OfERF112	evm.model.Contig313.4	225	25.05	5.23	68.53	-0.683	Nucleus
OfERF113	evm.model.Contig4.176	201	21.79	5.17	63.09	-0.531	Nucleus
OfERF114	evm.model.Contig4.ff1	354	40.15	4.97	55.55	-0.795	Nucleus
OfERF115	evm.model.Contig4.41	312	33.48	8.57	51.19	-0.651	Nucleus
OfERF116	evm.model.Contig4.52	359	40.15	5.68	42.93	-0.818	Nucleus
OfERF117	evm.model.Contig430.17	190	21.34	5.55	73.88	-0.650	Nucleus
OfERF118	evm.model.Contig47.42	347	39.07	4.77	45.04	-0.772	Nucleus
OfERF119	evm.model.Contig568.10	239	27.39	7.73	61.00	-0.740	Nucleus
OfERF120	evm.model.Contig568.11	224	24.91	5.23	67.38	-0.667	Nucleus
OfERF121	evm.model.Contig12.276	196	22.41	8.30	55.76	-0.545	Nucleus
OfERF122	evm.model.Contig12.319	213	23.82	8.55	55.22	-0.754	Nucleus
OfERF123	evm.model.Contig12.406	224	24.36	5.27	62.59	-0.396	Nucleus
OfERF124	evm.model.Contig145.41	239	26.54	5.30	61.17	-0.619	Nucleus
OfERF125	evm.model.Contig164.21	229	25.12	9.22	66.89	-0.789	Nucleus

OfERF126	evm.model.Contig21.17	321	35.82	4.85	37.23	-0.730	Nucleus
OfERF127	evm.model.Contig26.240	236	25.79	9.37	61.25	-0.700	Nucleus
OfERF128	evm.model.Contig26.241	159	17.45	9.52	55.84	-0.589	Mitochondria
OfERF129	evm.model.Contig260.58	239	26.74	5.03	47.77	-0.442	Nucleus
OfERF130	evm.model.Contig260.59	169	19.18	9.56	46.43	-0.358	Mitochondria
OfERF131	evm.model.Contig36.16	205	23.42	5.03	45.56	-0.832	Nucleus
OfERF132	evm.model.Contig139.173	277	30.75	6.67	56.58	-0.805	Nucleus
OfERF133	evm.model.Contig139.75	240	26.67	7.68	28.43	-0.566	Nucleus
OfERF134	evm.model.Contig143.45	259	28.30	9.16	63.85	-0.660	Nucleus
OfERF135	evm.model.Contig178.27	258	29.12	9.17	46.89	-0.649	Nucleus
OfERF136	evm.model.Contig256.104	292	32.92	4.83	37.28	-0.549	Nucleus
OfERF137	evm.model.Contig15.84	237	25.84	5.87	61.81	-0.381	Nucleus
OfERF138	evm.model.Contig158.50	191	21.78	5.68	40.54	-0.570	Chloroplast
OfERF139	evm.model.Contig158.51	221	24.90	5.84	33.30	-0.455	Chloroplast
OfERF140	evm.model.Contig241.46	258	28.26	6.77	55.87	-0.683	Nucleus
OfERF141	evm.model.Contig252.3	235	26.78	5.00	47.27	-0.757	Nucleus
OfERF142	evm.model.Contig253.23	158	17.18	9.46	35.69	-0.511	Chloroplast
OfERF143	evm.model.Contig38.102	107	11.86	10.43	46.25	-0.626	Nucleus
OfERF144	evm.model.Contig56.161	349	38.85	8.86	41.78	-0.685	Nucleus
OfERF145	evm.model.Contig89.201	131	14.93	5.68	54.33	-1.090	Nucleus
OfERF146	evm.model.Contig10.125	159	18.11	6.32	57.93	-0.699	Nucleus
OfERF147	evm.model.Contig10.15	375	41.62	5.01	37.89	-0.696	Nucleus
OfERF148	evm.model.Contig10.174	244	27.42	8.86	62.65	-0.883	Nucleus
OfERF149	evm.model.Contig10.183	125	13.93	10.15	54.79	-0.730	Nucleus
OfERF150	evm.model.Contig10.43	190	21.10	6.85	62.65	-0.686	Nucleus
OfERF151	evm.model.Contig130.94	330	36.95	5.00	52.73	-0.709	Nucleus
OfERF152	evm.model.Contig245.55	326	34.87	5.83	58.09	-0.578	Nucleus
OfERF153	evm.model.Contig248.10	246	27.28	5.77	56.06	-0.678	Nucleus
OfERF154	evm.model.Contig248.95	185	19.67	5.32	52.12	-0.421	Nucleus
OfERF155	evm.model.Contig248.96	207	23.07	4.72	60.80	-0.552	Nucleus
OfERF156	evm.model.Contig8.180	213	23.57	4.58	50.56	-0.508	Nucleus
OfERF157	evm.model.Contig8.284	220	25.11	8.37	66.55	-0.844	Nucleus

OfERF158	evm.model.Contig110.226	270	30.51	8.77	49.10	-0.659	Nucleus
OfERF159	evm.model.Contig150.17	252	28.43	5.15	45.02	-0.950	Nucleus
OfERF160	evm.model.Contig150.40	237	25.77	5.13	51.67	-0.421	Nucleus
OfERF161	evm.model.Contig150.73	197	21.92	4.40	47.99	-0.959	Nucleus
OfERF162	evm.model.Contig225.25	238	25.93	5.14	50.70	-0.414	Nucleus
OfERF163	evm.model.Contig242.45	212	23.81	5.38	53.86	-0.629	Nucleus
OfERF164	evm.model.Contig242.49	148	16.76	9.36	62.51	-1.019	Nucleus
OfERF165	evm.model.Contig270.96	245	27.52	6.24	57.94	-0.551	Nucleus
OfERF166	evm.model.Contig316.24	366	40.20	5.93	63.19	-0.654	Nucleus
OfERF167	evm.model.Contig316.25	361	39.89	6.77	62.85	-0.751	Nucleus
OfERF168	evm.model.Contig316.29	143	16.24	6.84	81.67	-0.581	Nucleus
OfERF169	evm.model.Contig383.13	284	31.98	5.25	68.46	-0.613	Nucleus
OfERF170	evm.model.Contig383.14	287	32.51	6.05	56.50	-0.533	Nucleus
OfERF171	evm.model.Contig397.44	182	21.06	8.76	50.50	-0.681	Nucleus
OfERF172	evm.model.Contig415.6	353	39.08	6.55	42.85	-0.608	Nucleus
OfERF173	evm.model.Contig99.149	228	25.88	5.30	54.42	-0.801	Nucleus
OfERF174	evm.model.Contig176.105	241	26.89	9.04	58.26	-0.549	Nucleus
OfERF175	evm.model.Contig176.29	252	27.65	6.46	57.98	-0.717	Nucleus
OfERF176	evm.model.Contig19.302	211	22.92	5.17	63.40	-0.436	Nucleus
OfERF177	evm.model.Contig299.20	386	42.63	4.63	43.21	-0.739	Nucleus
OfERF178	evm.model.Contig3.275	178	19.93	5.87	49.86	-0.824	Nucleus
OfERF179	evm.model.Contig326.18	164	17.99	9.35	36.49	-0.630	Mitochondria
OfERF180	evm.model.Contig326.50	185	20.37	8.87	49.22	-0.792	Nucleus
OfERF181	evm.model.Contig43.87	139	15.76	7.72	60.64	-0.820	Nucleus
OfERF182	evm.model.Contig720.1	164	17.99	9.35	36.49	-0.630	Mitochondria
OfERF183	evm.model.Contig115.73	260	28.01	6.50	49.38	-0.525	Nucleus
OfERF184	evm.model.Contig175.8	277	30.65	7.03	47.70	-0.765	Nucleus
OfERF185	evm.model.Contig200.45	244	26.77	8.18	43.11	-0.595	Nucleus
OfERF186	evm.model.Contig277.52	263	29.25	9.44	57.40	-0.545	Nucleus
OfERF187	evm.model.Contig490.37	270	29.47	8.52	39.26	-0.601	Nucleus
OfERF188	evm.model.Contig58.239	239	26.94	8.29	33.09	-0.659	Nucleus
OfERF189	evm.model.Contig121.39	282	31.95	5.62	61.69	-0.570	Nucleus

OfERF190	evm.model.Contig17.108	128	14.70	7.80	69.14	-0.988	Nucleus
OfERF191	evm.model.Contig17.110	213	23.98	4.85	60.95	-0.641	Nucleus
OfERF192	evm.model.Contig187.77	183	20.98	7.76	43.57	-0.674	Nucleus
OfERF193	evm.model.Contig234.20	303	34.22	8.75	38.42	-0.528	Nucleus
OfERF194	evm.model.Contig234.33	352	39.30	5.07	45.85	-0.593	Nucleus
OfERF195	evm.model.Contig234.73	232	24.96	5.13	67.22	-0.657	Nucleus
OfERF196	evm.model.Contig25.148	356	39.86	4.82	48.49	-0.691	Nucleus
OfERF197	evm.model.Contig25.213	232	26.10	5.17	42.98	-0.740	Nucleus
OfERF198	evm.model.Contig317.37	352	39.36	5.15	45.46	-0.585	Nucleus
OfERF199	evm.model.Contig317.48	303	34.18	8.25	39.15	-0.523	Nucleus
OfERF200	evm.model.Contig373.11	194	21.59	4.86	49.31	-0.830	Nucleus
OfERF201	evm.model.Contig373.54	236	25.60	5.74	47.06	-0.440	Nucleus
OfERF202	evm.model.Contig393.15	405	45.04	9.23	50.53	-0.527	Nucleus
OfERF203	evm.model.Contig465.4	377	41.40	8.59	67.39	-0.661	Nucleus
OfERF204	evm.model.Contig73.53	250	28.14	5.57	52.54	-0.614	Nucleus
OfERF205	evm.model.Contig79.26	208	23.97	6.62	41.35	-0.725	Nucleus
OfERF206	evm.model.Contig14.357	285	32.49	4.98	57.82	-0.716	Nucleus
OfERF207	evm.model.Contig2.157	250	27.56	6.85	56.87	-0.888	Nucleus
OfERF208	evm.model.Contig5.481	210	22.84	9.21	42.10	-0.469	Nucleus
OfERF209	evm.model.Contig77.131	342	37.96	5.58	68.55	-0.930	Nucleus
OfERF210	evm.model.Contig77.52	220	23.83	5.15	48.35	-0.127	Chloroplast
OfERF211	evm.model.Contig13.303	228	24.93	5.77	57.44	-0.686	Nucleus
OfERF212	evm.model.Contig172.33	160	17.67	9.46	51.25	-0.624	Mitochondria
OfERF213	evm.model.Contig18.101	232	25.89	5.71	46.24	-0.984	Nucleus
OfERF214	evm.model.Contig182.65	223	24.39	5.86	59.25	-0.471	Nucleus
OfERF215	evm.model.Contig228.45	227	24.49	5.54	46.56	-0.342	Nucleus
OfERF216	evm.model.Contig286.36	187	21.11	4.49	69.99	-1.061	Nucleus
OfERF217	evm.model.Contig323.12	227	24.51	5.54	46.56	-0.324	Chloroplast
OfERF218	evm.model.Contig341.6	259	27.71	6.44	53.58	-0.417	Nucleus
OfERF219	evm.model.Contig44.160	392	43.25	6.96	49.05	-0.550	Nucleus
OfERF220	evm.model.Contig233.54	177	18.84	5.04	63.74	-0.310	Chloroplast
OfERF221	evm.model.Contig266.42	220	24.69	7.01	49.21	-0.611	Nucleus

OfERF222	evm.model.Contig266.46	222	24.96	7.02	48.52	-0.637	Nucleus
OfERF223	evm.model.Contig386.58	220	24.78	7.78	51.09	-0.653	Nucleus
OfERF224	evm.model.Contig386.63	222	24.93	6.60	49.89	-0.603	Nucleus
OfERF225	evm.model.Contig159.22	321	35.24	8.97	48.37	-0.545	Nucleus
OfERF226	evm.model.Contig159.96	375	40.47	7.88	71.24	-0.583	Nucleus
OfERF227	evm.model.Contig24.1	412	45.65	8.82	48.07	-0.685	Nucleus
OfERF228	evm.model.Contig24.94	217	24.56	5.20	49.37	-0.769	Nucleus
OfERF229	evm.model.Contig24.96	143	16.20	5.59	62.43	-1.017	Nucleus
OfERF230	evm.model.Contig24.97	129	14.74	5.67	52.53	-0.936	Nucleus
OfERF231	evm.model.Contig7.104	328	35.42	5.73	55.40	-0.452	Nucleus
OfERF232	evm.model.Contig7.297	161	17.53	9.59	45.53	-0.663	Nucleus
OfERF233	evm.model.Contig714.4	175	19.05	5.08	57.86	-0.521	Nucleus
OfERF234	evm.model.Contig324.87	194	21.76	8.57	68.73	-0.491	Nucleus
OfERF235	evm.model.Contig324.88	195	22.08	5.61	66.39	-0.584	Nucleus
OfERF236	evm.model.Contig324.89	158	17.90	9.93	57.31	-0.601	Nucleus
OfERF237	evm.model.Contig351.42	153	17.34	5.07	48.46	-0.549	Nucleus
OfERF238	evm.model.Contig351.44	137	15.15	5.86	61.56	-0.701	Nucleus
OfERF239	evm.model.Contig351.48	164	18.25	4.91	62.38	-0.491	Nucleus
OfERF240	evm.model.Contig57.52	215	24.04	5.73	56.56	-0.507	Nucleus
OfERF241	evm.model.Contig57.53	125	14.06	6.14	58.96	-0.935	Nucleus
OfERF242	evm.model.Contig57.54	131	14.65	7.87	40.00	-1.070	Nucleus
OfERF243	evm.model.Contig62.211	204	22.43	7.90	32.71	-0.755	Nucleus
OfERF244	evm.model.Contig62.230	151	17.30	9.84	57.45	-0.648	Nucleus
OfERF245	evm.model.Contig62.231	195	22.00	5.96	65.50	-0.568	Nucleus
OfERF246	evm.model.Contig62.232	194	21.86	8.25	69.72	-0.483	Nucleus
OfERF247	evm.model.Contig75.23	141	15.92	9.19	51.72	-0.811	Nucleus
OfAP2-1	evm.model.Contig167.175	361	40.82	8.27	44.59	-0.825	Nucleus
OfAP2-2	evm.model.Contig215.28	378	41.47	9.17	47.10	-0.425	Nucleus
OfAP2-3	evm.model.Contig349.99	395	44.64	9.09	55.07	-0.707	Nucleus
OfAP2-4	evm.model.Contig71.172	518	58.42	6.91	51.80	-0.809	Nucleus
OfAP2-5	evm.model.Contig71.71	633	70.43	6.39	51.90	-0.793	Nucleus
OfAP2-6	evm.model.Contig45.165	394	44.74	9.41	60.37	-0.859	Nucleus
OfAP2-7	evm.model.Contig180.58	332	37.82	6.09	51.48	-0.714	Nucleus

OfAP2-8	evm.model.Contig37.71	469	51.72	6.76	45.11	-0.611	Nucleus
OfAP2-9	evm.model.Contig421.24	535	59.68	5.90	49.57	-0.756	Nucleus
OfAP2-10	evm.model.Contig122.40	650	72.15	6.03	49.33	-0.759	Nucleus
OfAP2-11	evm.model.Contig488.10	372	42.63	6.77	51.93	-0.754	Nucleus
OfAP2-12	evm.model.Contig488.22	649	72.03	6.03	50.11	-0.729	Nucleus
OfAP2-13	evm.model.Contig160.46	455	51.05	6.19	52.52	-0.785	Nucleus
OfAP2-14	evm.model.Contig259.114	575	63.00	5.46	41.79	-0.701	Nucleus
OfAP2-15	evm.model.Contig287.31	372	42.83	8.13	56.56	-0.799	Nucleus
OfAP2-16	evm.model.Contig173.118	457	49.74	6.18	46.45	-0.535	Nucleus
OfAP2-17	evm.model.Contig355.19	462	50.46	6.76	50.20	-0.540	Nucleus
OfAP2-18	evm.model.Contig59.19	539	60.07	6.14	50.78	-0.751	Nucleus
OfAP2-19	evm.model.Contig103.14	609	67.72	6.60	51.46	-0.714	Nucleus
OfAP2-20	evm.model.Contig120.63	363	41.11	9.25	57.13	-0.636	Nucleus
OfAP2-21	evm.model.Contig10.33	487	54.72	6.41	49.77	-0.827	Nucleus
OfAP2-22	evm.model.Contig157.17	329	38.01	8.30	54.19	-0.829	Nucleus
OfAP2-23	evm.model.Contig165.67	499	55.37	5.95	50.97	-0.636	Nucleus
OfAP2-24	evm.model.Contig249.22	419	47.23	8.64	49.09	-0.869	Nucleus
OfAP2-25	evm.model.Contig258.50	457	49.91	7.66	38.95	-0.598	Nucleus
OfAP2-26	evm.model.Contig110.183	362	40.50	9.35	43.75	-0.695	Nucleus
OfAP2-27	evm.model.Contig124.19	578	64.57	6.80	42.45	-0.644	Nucleus
OfAP2-28	evm.model.Contig153.19	578	64.56	6.64	41.90	-0.638	Nucleus
OfAP2-29	evm.model.Contig144.54	395	44.39	8.93	51.39	-0.640	Nucleus
OfAP2-30	evm.model.Contig3.342	494	54.91	6.11	47.33	-0.637	Nucleus
OfAP2-31	evm.model.Contig326.22	504	56.16	6.55	58.16	-0.947	Nucleus
OfAP2-32	evm.model.Contig85.142	609	67.58	6.39	51.21	-0.674	Nucleus
OfAP2-33	evm.model.Contig523.11	551	61.79	9.16	46.84	-0.683	Nucleus
OfAP2-34	evm.model.Contig79.155	529	58.61	5.77	48.21	-0.741	Nucleus
OfAP2-35	evm.model.Contig154.100	684	75.49	5.71	50.22	-0.738	Nucleus
OfAP2-36	evm.model.Contig2.199	340	38.85	6.50	47.15	-0.974	Nucleus
OfAP2-37	evm.model.Contig5.236	362	40.87	8.88	45.12	-0.842	Nucleus
OfAP2-38	evm.model.Contig575.3	681	75.12	5.67	50.55	-0.749	Nucleus
OfAP2-39	evm.model.Contig113.63	465	51.68	7.06	35.01	-0.639	Nucleus
OfAP2-40	evm.model.Contig117.167	469	51.46	7.99	47.31	-0.579	Nucleus

OfAP2-41	evm.model.Contig7.414	370	42.47	9.22	56.78	-0.764	Nucleus
OfAP2-42	evm.model.Contig82.35	640	71.34	6.77	51.20	-0.715	Nucleus
OfAP2-43	evm.model.Contig35.59	110	12.79	9.77	72.89	-0.930	Nucleus
OfAP2-44	evm.model.Contig207.41	426	47.39	8.64	51.23	-0.786	Nucleus
OfAP2-45	evm.model.Contig26.50	432	47.56	5.86	50.11	-0.672	Nucleus
OfRAV1	evm.model.Contig206.48	342	38.45	8.98	37.56	-0.627	Nucleus
OfRAV2	evm.model.Contig460.28	361	40.66	5.61	37.03	-0.573	Nucleus
OfRAV3	evm.model.Contig61.117	360	40.51	9.06	49.28	-0.576	Nucleus
OfRAV4	evm.model.Contig61.39	346	38.82	9.75	39.53	-0.712	Nucleus
OfRAV5	evm.model.Contig279.75	350	39.22	7.65	41.66	-0.594	Nucleus
OfSOLO1	evm.model.Contig359.33	230	26.21	9.40	58.15	-0.776	Nucleus
