# Supplementary material for: Analysis of the Aging-Related AP2/ERF Transcription Factor Gene Family in Osmanthus fragrans
Source: Int J Mol Sci. 2024 Jul 23;25(15):8025. doi: 10.3390/ijms25158025 (PMC11312093; doi:10.3390/ijms25158025)
Supplement: Supplementary file 1 [file ijms-25-08025-s001.zip › Table S2.pdf]

Table S2. The gene ID of selected genes

**Number**	**Gene ID**	**Name shown in this paper**
1	evm.model.Contig105.116-gene	*OfERF61a*
2	evm.model.Contig135.89-gene	*OfERF4a*
3	evm.model.Contig164.21-gene	*OfERF3*
4	evm.model.Contig170.10-gene	*OfERF5a*
5	evm.model.Contig178.27-gene	*OfERF109a*
6	evm.model.Contig234.20-gene	*OfERF61b*
7	evm.model.Contig248.95-gene	*OfERF16*
8	evm.model.Contig248.96-gene	*OfERF17*
9	evm.model.Contig316.25-gene	*OfERF110a*
10	evm.model.Contig317.48-gene	*OfERF61c*
11	evm.model.Contig33.24-gene	*OfERF26*
12	evm.model.Contig35.282-gene	*OfERF106*
13	evm.model.Contig38.209-gene	*OfERF110b*
14	evm.model.Contig38.70-gene	*OfERF1-3*
15	evm.model.Contig383.14-gene	*OfERF5b*
16	evm.model.Contig490.37-gene	*OfERF109b*
17	evm.model.Contig5.481-gene	*OfERF4b*
18	evm.model.Contig58.239-gene	*OfERF109c*
19	evm.model.Contig64.26-gene	*OfERF61d*
20	evm.model.Contig78.178-gene	*OfERF4c*
