# Supplementary material for: Analysis of the Aging-Related AP2/ERF Transcription Factor Gene Family in Osmanthus fragrans
Source: Int J Mol Sci. 2024 Jul 23;25(15):8025. doi: 10.3390/ijms25158025 (PMC11312093; doi:10.3390/ijms25158025)
Supplement: Supplementary file 1 [file ijms-25-08025-s001.zip › Table S3.pdf]

Table S3. Primer sequences used for qRT-PCR

Number	Gene name	Primer sequence
1	*OfERF61a*	F:GATCCCATCTTTCGACCCG
		R:ACTGGCAAGAACTTCCCAAATC
2	*OfERF4a*	F:CCGTGGAGTCATCAAGAGCC
		R:CCGCCGTTGGGAAAATG
3	*OfERF3*	F:GCGGCAGCAAGGCAAAT
		R:CCTTAGATCCACCAGATCCGTTA
4	*OfERF5a*	F:GAGGGAGCAAAGCAATAGTGAA
		R:CGGAGGGTTGGAGTTGGTAA
5	*OfERF109a*	F:GACTCCGCCGCTTCTCATT
		R:GCAACCGATAACAACACCTTCA
6	*OfERF61b*	F:ATTACGAAAACCAAACGAACCC
		R:TCGCTGCCTCACTCCTCTGT
7	*OfERF16*	F:TTTGCTTGCGTGGTCCTACA
		R:TCCCAACCCAGTCGAGTCAG
8	*OfERF17*	F:CGCCACCGGAGATAGTCAA
		R:GGATAAAACCCGAAATCAGCA
9	*OfERF110a*	F:TCAAGGTGGCGAATCAAGG
		R:CGGAAAATAGATGACTCGGTTG
10	*OfERF61c*	F:CCAGTCCCCGAGACATCAGA
		R:TGTCGCTGCCTCACTCCTCT
11	*OfERF26*	F:GCATCTCAAACTGCCACCG
		R:CCAGCCTGGAATATGCTCACT
12	*OfERF106*	F:CTGGTCGGGCTCGTATTCTT
		R:GTTTCATCGGCGTATTCGGT
13	*OfERF110b*	F:GTCGTTTGCTGGTGGTACTGG
		R:TGCTCGTTGTTTCTGCTTGTG
14	*OfERF1-3*	F:ACAGGAGGGATCTCAGTTTTGC

		R:AGCTCGAACATCCAACTGGTAA
15	*OfERF5b*	F:AGCGGAGGTAGTGGTGATGG
		R:GCTGTGGATAACCGAAAGGAG
16	*OfERF109b*	F:CGGCTTACGAGTGAAGAGGAA
		R:TGGCGGTGTCACCAGTTATG
17	*OfERF4b*	F:GGCTAATGGGATAAAGGGAGG
		R:TACGCGGGTCACGGATTT
18	*OfERF109c*	F:AGTTTTCGGCGGTTTCCAG
		R:AGTTGCATCCCAAACACCCT
19	*OfERF61d*	F:GATGGGTTTTGGAAGGGTGA
		R:GGAAACCGAGGAAGGTGAATT
20	*OfERF4c*	F:TTCCATTCCAGAACCACCATT
		R:CGGGAAATCTCCGACGAAC
21	*OfACT*	F:CCCAAGGCAAACAGAGAAAAAT
		R:ACCCCATCACCAGAATCAAGAA
