# Supplementary material for: Analysis of the Aging-Related AP2/ERF Transcription Factor Gene Family in Osmanthus fragrans
Source: Int J Mol Sci. 2024 Jul 23;25(15):8025. doi: 10.3390/ijms25158025 (PMC11312093; doi:10.3390/ijms25158025)
Supplement: Supplementary file 1 [file ijms-25-08025-s001.zip › Table S4.pdf]

Table S4. AP2/ERF transcription factor subfamily classification

**Subfamily**	**Structure**
AP2	AP2/AP2
ERF	AP2
DREB	AP2
RAV	AP2/B3
Soloist	AP2
